# The effectiveness of using virtual reality technology for perioperative anxiety among adults undergoing elective surgery: a randomised controlled trial protocol

**DOI:** 10.1186/s13063-022-06908-3

**Published:** 2022-12-02

**Authors:** Salihah Asiri, Michelle Guilhermino, Jed Duff

**Affiliations:** 1 School of Nursing, Umm Al-Qura University, Makkah, Saudi Arabia; 2 School of Nursing, Faculty of Health, Queensland University of Technology, Brisbane, Australia; 3 Australian College of Perioperative Nurses (ACORN), QLD, Australia; 4 Nursing & Midwifery Research Centre, Centre for Clinical Nursing, Royal Brisbane & Women’s Hospital, Building 34, Level 5, Herston, QLD 4029 Australia; 5 School of Nursing and Midwifery, College of Health, Medicine and Wellbeing, University of Newcastle, Callaghan, Australia; 6 John Hunter Hospital – Intensive care Services, Newcastle, Australia

**Keywords:** Perioperative anxiety, Elective surgery, Virtual reality, Technology, Randomised control trial

## Abstract

**Background:**

More than 2.5 million people are admitted for surgery in Australia each year, and between 40 to 80% will experience moderate to high preoperative anxiety. Elevated levels of preoperative anxiety can increase the risk of postoperative complications such as pain, delayed wound healing, infection, prolonged recovery, and longer hospitalisation. Limited previous research on Virtual Reality (VR) indicates a positive impact on surgery-related anxiety and suggests that the intervention potentially leads to reduce postoperative complications.

**Objective:**

To evaluate the effectiveness of using VR technology for perioperative anxiety among adults undergoing elective surgery.

**Method:**

A two-group parallel randomised controlled trial (RCT) will be conducted, including 150 adult patients (aged 18 years and over) undergoing elective surgery and requiring an overnight stay at a major metropolitan hospital. Eligible participants will be screened for anxiety via the Amsterdam Preoperative Anxiety and Information score (APAIS). Those with moderate to severe anxiety will be randomly allocated to receive the VR session or usual care, in the preoperative holding area. Intervention participants will use a head-mounted VR device to watch and listen to a nature scene for 10 minutes.

**Study outcomes:**

The primary outcome is perioperative anxiety measured using the visual analogue scale for anxiety (VAS-A). Secondary outcomes include stress levels (measured by saliva cortisol level and heart rate), postoperative pain, patient satisfaction with perioperative care, hospital length of stay, and VR-associated adverse events.

**Conclusion:**

This study will help evaluate if a brief preoperative VR session can reduce perioperative anxiety for adult elective surgical patients.

**Trial registration:**

Australia and New Zealand Clinical Trials Registry (ANZCTR) ACTRN12620001350910.

**Supplementary Information:**

The online version contains supplementary material available at 10.1186/s13063-022-06908-3.

## Administrative information

Note: the numbers in curly brackets in this protocol refer to SPIRIT checklist item numbers. The order of the items has been modified to group similar items (see http://www.equator-network.org/reporting-guidelines/spirit-2013-statement-defining-standard-protocol-items-for-clinical-trials/).

**Table:** 

Title	The effectiveness of using virtual reality technology for perioperative anxiety among adults undergoing elective surgery: A randomised controlled trial protocol
Trial registration	Australia and New Zealand Clinical Trials Registry (ANZCTR) ACTRN12620001350910.
Protocol version	Version 2 16.06.2021
Funding	Australian College of Perioperative Nurses (ACORN)
Author details	a School of Nursing, Umm Al-Qura University, Makkah, Saudi Arabia b School of Nursing, Faculty of Health, Queensland University of Technology, Brisbane, Australia c Australian College of Perioperative Nurses (ACORN) e School of Nursing and Midwifery, College of Health, Medicine and Wellbeing, University of Newcastle, Australia f John Hunter Hospital – Intensive care Services, Newcastle, Australia
Name and contact information for the trial sponsor	Salihah Asiri, Address: Nursing & Midwifery Research Centre, Centre for Clinical Nursing, Building 34, Level 5, Royal Brisbane & Women’s Hospital, Herston, QLD, 4029, Australia Mobile: +61 412109444 Email: Salihah.asiri@hdr.qut.edu.au
Role of sponsor	The sponsors had no role in collection, analysis and interpretation of data, and had no role in writing the report, and in the decision to submit this article for publication.

## Background {6a}

Rates of anxiety tend to increase among patients who are scheduled for surgery, regardless of the surgery type. The prevalence of preoperative anxiety has been estimated by a number of studies worldwide [1–3]. A prospective study conducted in Austria, Canada, and Sri Lanka revealed that 45.3%, 89%, and 76.7% of surgical patients respectively experienced significant preoperative anxiety [1–3].

About 40 to 80% of adult surgical patients report experiencing anxiety triggered by different causes [4–6]. These include pain anticipation, loss of independence, separation from family, fear of the anaesthesia, fear of unfavourable diagnosis, changes in appearance, or the possibility of death [7, 8]. Most patients experience anxiety during the preoperative phase, and it is usually recognised as a normal patient response. The extent to which each patient manifests anxiety depends on many variables, such as age, gender, previous surgical experiences, educational status, type of surgery, current health status, and socioeconomic status [9]. Some patients, such as women or young patients, have high levels of preoperative anxiety [9, 10].

High or prolonged levels of preoperative anxiety lead to a number of negative consequences [7]. The most common postoperative complaint is postoperative pain [11, 12]. As a result, the need for postoperative pain medication increases, and this affects postoperative recovery [11, 12]. A study by Kavakci et al. [11] investigated the relationship between the level of preoperative anxiety and the pain level in the postoperative period, analgesic requirements, and the duration of hospital stay among adult patients (*n* = 103) who underwent ear, nose, and throat (ENT) surgery. There was a medium positive correlation between a patient’s anxiety level based on the Hospital Anxiety and Depression Scale, and their postoperative pain based on the visual analogue scale for pain (VAS-P) scores (*r* = 0.30, *p* < 0.002) [11]. In a recent study, preoperative anxiety and postoperative pain were examined among Chinese patients undergoing laparoscopic hysterectomy. Results show that patients with higher preoperative anxiety experience more postoperative pain [13].

As anxiety raises cortisol levels, preoperative anxiety has also been shown to delay wound healing, cause imbalanced fluid and electrolyte levels, and increase the risk of infection [14, 15]. In an experimental study, 24 healthy young men undergoing 4-mm punch biopsy were monitored for 21 days using ultrasound biomicroscopy to evaluate wound healing [16]. Self-reported stress levels were evaluated using the Perceived Stress Scale (PSS) self-report questionnaire. The results showed a significant negative correlation between speed of wound healing and the Perceived Stress Scale (PSS) scores (*r* = −.59; *p* < .01), indicating that slower wound healing was associated with higher perceived stress on the biopsy day. Cortisol levels were significantly increased for both groups (*F* = 14, 71, *p* < 0.01), but the total cortisol levels of the ‘slow healing’ group were significantly higher than those of the ‘quick healing’ group (significant group effect: *F* = 5.60, *p* < 0.05) [16].

Many strategies have been used to manage high levels of preoperative anxiety in both inpatient and outpatient settings. Pharmacological interventions including anxiolytic drugs such as midazolam, fentanyl, morphine, and ketamine are most commonly utilised [17]. Pharmacological treatments can lead to adverse effects such as breathing difficulties, drowsiness, interference with anaesthesia drugs, and prolonged recovery [18]. So non-pharmacological interventions including music, educational videos, psychological counselling, and social support are increasingly common [7, 9, 19–21].

Recently, there is growing interest in reducing preoperative anxiety through virtual reality (VR) technology. VR has been defined as ‘a computer-generated display that allows or compels the user (or users) to have a sense of being present in an environment other than the one they are actually in and to interact with that environment’ [22]. It has been proposed that this immersive technology can be used as a distraction tool to deflect patients’ attention away from their concerns regarding their upcoming surgery [23–26]. Previous studies have measured the effectiveness of using VR for perioperative anxiety in children. Four previous studies have focused on VR for reducing perioperative anxiety in adult patients undergoing surgical procedures [23–26].

These studies show that VR significantly reduced anxiety compared to control groups while increasing patient satisfaction and self-reported preparedness for surgery. The length of hospital stay and pain level showed no significant change between VR and control groups [23–26]. A limitation of these studies was the inclusion of all surgical patients regardless of their preoperative anxiety level. The other limitations of these studies include failure to report adverse VR symptoms, such as nausea, vomiting, headaches, or discomfort. Also, none of the RCTs had used biomarkers for stress levels as an objective measurement tool [23–26].

This study aims to evaluate the effectiveness of using VR technology for perioperative anxiety among adults undergoing elective surgery who experiencing moderate levels of anxiety. Secondary outcomes that will be evaluated include pain, stress, patient satisfaction with perioperative care, length of hospital stay, and VR-related adverse effects.

## Methods

### Study design and setting {8} {9}

**Fig. 1:**
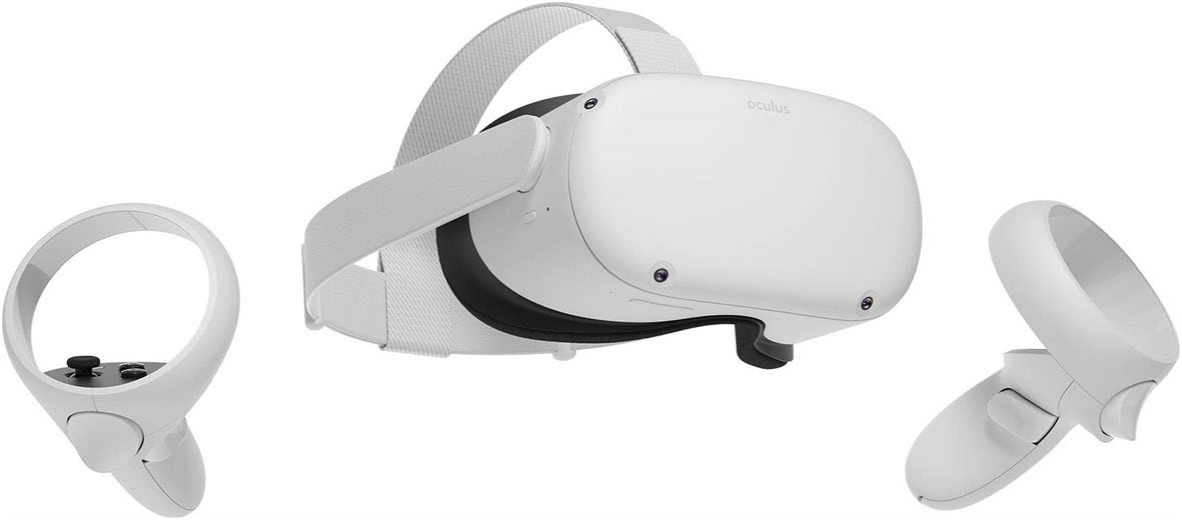
Oculus Quest 2 Virtual Reality Headset™

A two-group parallel, single-centre, superiority, randomised controlled trial (RCT) with an allocation ratio of 1:1 will be conducted and reported in accordance with the CONSORT guidelines [27]. A total of 150 patients undergoing elective surgery will be randomised to the control (standard preoperative care) or intervention (standard preoperative care plus VR intervention) groups. The intervention will be delivered using the Oculus Quest 2 Virtual Reality Headset™ fitted with a smartphone (Fig. 1). This device has been chosen because it is the most advanced commercially available all-in-one VR device from Oculus, equipped with a highly immersive headset, two controllers, and integrated headphones. This study will be conducted in the surgical unit of a major Australian metropolitan hospital.

### Study aim {7}

The aim of this study is to evaluate the effectiveness of using VR technology for perioperative anxiety among adults undergoing elective surgery.

### Hypothesis {6b}

Compared with standard care, a VR intervention delivered during the preoperative period will significantly reduce perioperative anxiety in adult elective surgical patients by 20 points on a 100-point visual analogue scale for anxiety (VAS-A).

### Recruitment {15} {26a}

The primary investigator will consult the hospital’s admission team to review the surgical list and identify potential participants. The potential participants will be asked to participate in the study via the hospital confirmation SMS providing them details of their surgery. They will be provided with a participant information statement and consent form via a link in the SMS. Potential participants will be requested to return their signed consent prior to their day of surgery if they are interested in participating in the study.

### Inclusion and exclusion criteria {10}

Patients will be eligible for inclusion if they are 18 years old and over, scheduled for elective surgery, can understand English, and can follow instructions. Patients will be excluded if they are scheduled for day surgery, have a history of seizures or motion sickness, have visual impairment, or are unable to complete a self-reported questionnaire.

### Screening

Patients who meet the above inclusion criteria will be screened for their anxiety level using a validated tool—the Amsterdam Preoperative Anxiety and Information score (APAIS) [28, 29]—on the day of their surgery in the preoperative holding area. Patients with moderate to high preoperative anxiety levels—described by preoperative anxiety score ≥11 [30]—will be included in the study and randomised to the intervention or control group.

The APAIS is a rapid and clinically practical assessment tool developed by Nelly Moerman and previously validated against other scales to evaluate patients’ preoperative anxiety with good sensitivity and strong specificity for clinically significant anxiety [28, 29]. It consists of six questions in total. Each question is rated on a 5-point Likert scale from “not at all” to “extremely”. The sum of scores from questions 1, 2, 4, and 5 show the anxiety level, while the sum of scores from questions 3 and 6 show the level of information required by each individual. A patient with a score of 11 or more (scoring range from 4 to 20) on the anxiety scale experiences anxiety and requires further intervention [28, 29, 31]. On the need for information scale, patients scoring 2–4 are categorised as having minimal or no need for information, patients scoring 5–7 are categorised as having an average need for information, and patients scoring 8–10 are considered as having high need for information [28, 31].

### Randomisation and allocation concealment {16a} {16b}{16c}

Participants will be randomly allocated to intervention or control via block randomisation on a 1:1 ratio (control: intervention) using a computer-generated random number sequence created by an independent statistician. In addition, randomisation will be stratified by surgery type (i.e. ENT surgery, general surgery, neurosurgery, maxillofacial surgery, urology surgery, vascular surgery, orthopaedics, and other surgeries). Electronic allocation via an online platform (the REDCap™ randomisation module) will be used to conceal the treatment allocation from the researcher until they have been deemed eligible and screened.

### Blinding {17b}

The Research Assistant involved in outcome data collection will be blinded to group allocation, as will the statistician who will conduct the data analysis. Clinicians (nurses, doctors, and other health providers) will not be told who is participating in the study nor their group allocation; however, it is acknowledged that it is not possible to prevent participants from sharing this information with them.

### Intervention and control {11a)

The control group will receive standard preoperative care only, while the intervention group will receive standard preoperative care plus VR. Standard preoperative care will include preparing the preoperative surgical site, preoperative bathing, preoperative hair removal, wearing theatre gown, removing all jewellery, correct patient ID bracelet, and completing the patient preoperative checklist and health record.

Standard preoperative care currently does not include routine anxiety screenings and nonpharmacological and pharmacological interventions.

The trial site has more than 17 chairs in the holding area where patients can wait to be called. In that area, there is TV and magazines available upon request.

### Virtual reality intervention {11a}

**Fig. 2:**
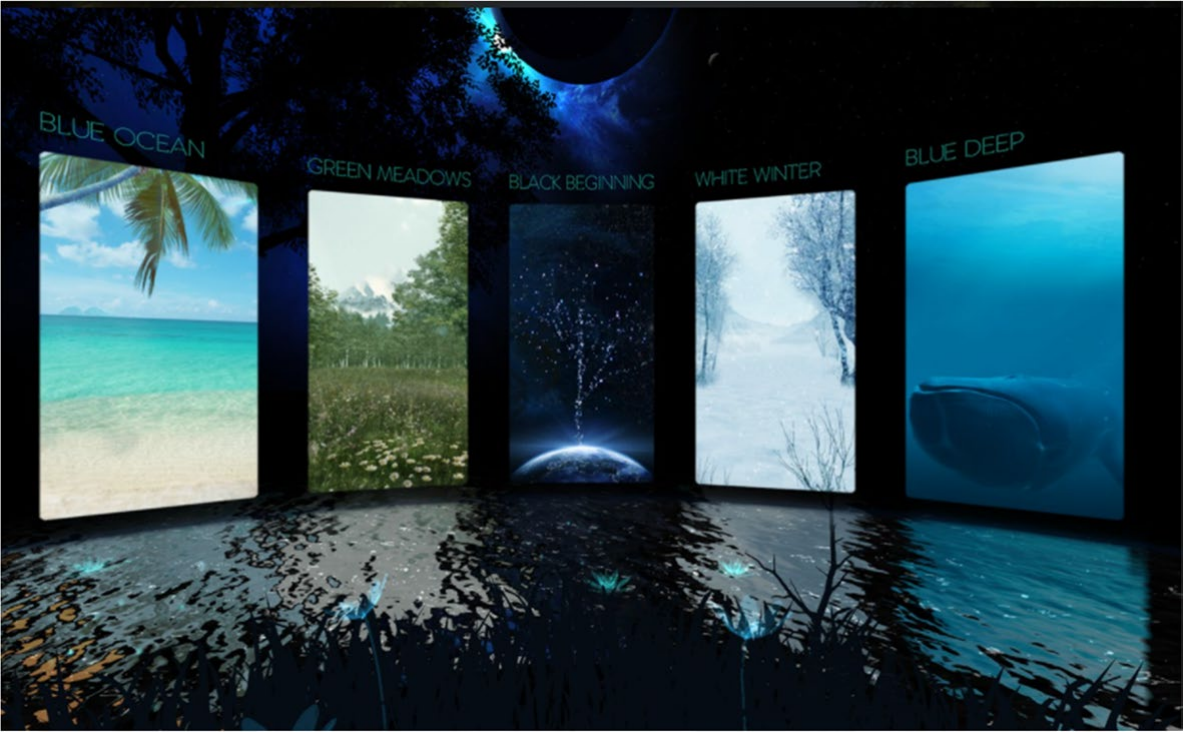
Virtual reality scenes retrieved from https://www.oculus.com/experiences/quest/2616537008386430/

The participants will be asked to wear the VR headset for 10 min, which has been suggested in the literature to be the optimal time frame for VR without adverse side effects [32, 33]. The participants will be seated in the preoperative holding area while immersed in a VR. The preoperative holding area has been selected for administration of the intervention because evidence suggests preoperative anxiety levels among adult surgical patients peak in the preoperative holding area [34]. The VR will be used for relaxation purposes with nine natural scenes combined with natural sounds and music, for the participant to select from: blue moon, red savanna, blue ocean, green meadows, black beginning, white winter, blue deep, red fall, and the orange sunset (Fig. 2). The participant’s face and forehead will be cleaned using skin-friendly antibacterial cleaning wipes before using the VR device. Disposable hygiene covers will be used to protect the VR device. The researcher will provide the intervention and monitor the participant for adverse side effects such as headache, nausea, vomiting, sweating, fatigue, drowsiness, disorientation, and apathy throughout the session [33].

### Criteria for discontinuing the trial in an individual {11b}

The researchers will monitor the participants continuously for side effects. If a side effect occurs, the VR session will be ceased immediately, and a treating medical team will be consulted. Also, there may be a risk of exacerbating the participant’s anxiety level associated with using a survey to assess anxiety. If this is the case, participants to be instructed to stop the survey and appropriate support will be provided by the researcher or medical team.

### Strategies to improve adherence to interventions {11c}

This study includes one-time intervention during the study and is a pragmatic study. The researcher will provide the intervention and the research assistant will collect the data. Researchers will daily check the data in the REDCap™ platform. Also, all researchers share information through periodic meetings (weekly) and discuss the study and appropriate management when problems occur.

### Concomitant care and interventions that are permitted or prohibited during the trial {11d}

There is no care or intervention prohibited during the trial. But we anticipate that some participants may regularly use anti-anxiety/anti-depressant medications. If this is the case, the research assistant will document the name and dose of the medication taken prior surgery.

### *Outcomes {12}*

#### Primary outcome

Perioperative anxiety will be assessed by the Research Assistant at three time points: on admission to the preoperative area (T1), immediately before surgery (T2), and 1 h on arrival to the post-anaesthesia care unit (T3). Participants will be asked to rate their level of anxiety using the visual analogue scale for anxiety (VAS-A). The VAS-A is valid, reliable, and frequently used to evaluate perioperative anxiety levels in surgical populations [35]. The VAS-A consists of a 100-mm horizontal line representing two behavioural extremes at either end of the continuum (i.e., ‘not at all anxious’ = 0, whereas ‘extremely anxious’ = 100). Scores on the VAS-A of 25 or higher reflect significant levels of anxiety [35].

**Figure:**
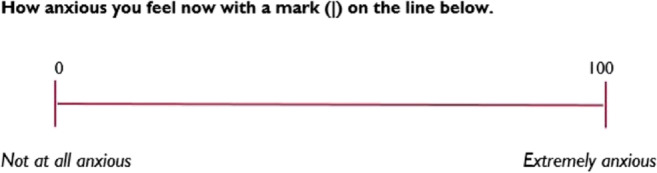
Visual analog scale for anxiety (VAS-A)

### Secondary outcomes

**Fig. 3:**
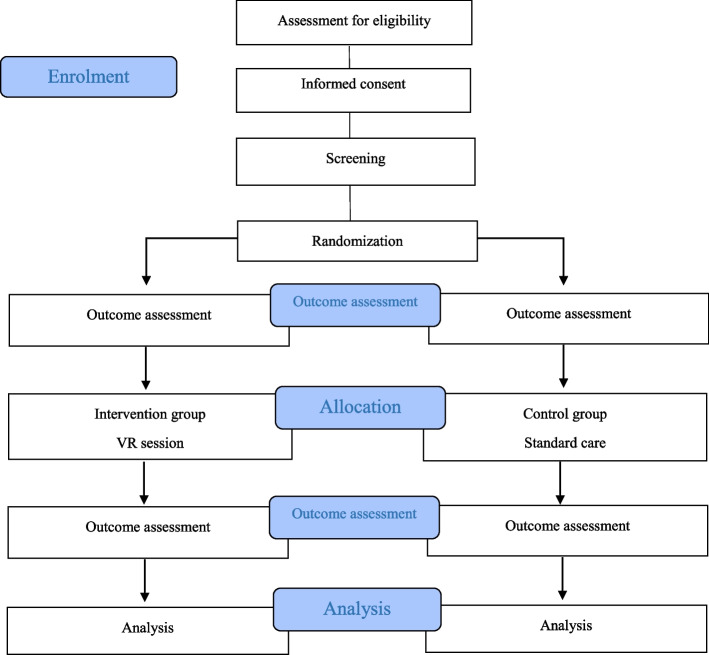
Study flow diagram

The secondary outcomes to be assessed include:

1. Stress levels will be assessed via two methods:
  A) Saliva cortisol levels as measured by collecting 4–5 ml of saliva in a clean glass tube. The samples will be labelled with participant ID number and stored at −18°C until they are transferred to Saliva and Liquid Biopsy Translational Laboratory, Queensland University of Technology, for analysis using the Cortisol ELISA Assay Kit™ by an independent laboratory. The analysis results will be intended to determine the quantitative of cortisol by an enzyme immunoassay in human saliva. This is a highly sensitive test, with results changing in minutes after exposure to an anxiety-inducing or relaxing stimulus [26]. The sample will be used for the purposes of this research project only and will be destroyed at the end of this project.
    Saliva samples will be collected from participants by the Research Assistant at two-time points: on admission to the preoperative area (T1) and immediately before surgery (T2).
  B) Heart rate (HR) as measured manually for 1 min and the mean HR will be calculated. The Research Assistant will record HR at three time points: on admission to the preoperative area (T1), immediately before surgery (T2), and 1 h on arrival to the post-anaesthesia care unit (T3).
2. Pain level as measured by the visual analogue scale for pain (VAS-P). The Research Assistant will ask the participants to rate their pain on the VAS-P tool at two time points: on admission to the preoperative area (T1) and 1 h on arrival to the post-anaesthesia care unit (T3).
3. Patient satisfaction as measured by the Leiden Perioperative Patient Satisfaction questionnaire (LPPSq). The questionnaire is a valid and reliable tool used to assess and measure different aspects of patient satisfaction with perioperative care with 39 questions [36]. The participants will be asked to complete the questionnaire 24 h after surgery before discharge (T4).
4. Hospital length of stay (LOS) as measured from the date of admission to discharge from hospital. The Research Assistant will collect this data from administrative records post-discharge.
5. Adverse effects of the VR intervention at any time point as measured by the Virtual Reality Symptom Questionnaire (VRSQ). The questionnaire is a valid and reliable measure used to evaluate the possible occurrence of cybersickness symptoms, a type of motion sickness caused by exposure to VR [37]. The researcher will ask the participants in the intervention group to complete the questionnaire immediately before surgery (T2) (Fig. 3, Table 1).

**Table 1:** SPIRIT flow diagram

Study period							
	Pre-admission	Preoperative phase			Postoperative phase		Discharge
Timepoint^a^	−t_1_	t_0_	t_1_	t_2_	t_3_	t_4_	t_5_
Enrolment:							
Eligibility	X						
Informed consent	X						
Screen Anxiety level		X					
Allocation		X					
Interventions:							
VR exposure				X			
Assessments:							
**Primary outcome:**			X	X	X		
Perioperative anxiety							
**Secondary outcomes**:			X	X	X		
Stress level							
Pain			X		X		
Patient satisfaction						X	
Length of hospital stay							X
VR adverse effect				X			

^a^−t_1_, pre-admission; t_0_, on admission to hospital; t_1_, on admission to preoperative area; t_2_, immediately before surgery (holding area); t_3_, 1 h post-surgery; t_4_, after 24 h post-surgery and before discharge; t_5_, post-discharge

### Other data

Other data will include age, sex, surgery type, type and dose of anaesthetic, anti-anxiety medications, history of previous surgeries, and discharge data. This data will be collected from the participant’s health record by the researcher.

### Sample size {14}

Based on the previous study by Bekelis et al. [24], a sample of 150 participants (75 per group) will provide 80% power to detect a 20% change on the 100-point VAS-A between groups with a type 1 error rate of 5%. This calculation assumes a dropout rate of 15%.

### Statistical plan {20a}

Characteristics of groups will be summarised using counts and percentages for categorical variables and means and standard deviations for continuous variables. The primary outcome (anxiety at Time 2 and Time 3 in stacked format) will be compared between groups at each follow-up time point using a linear mixed-effects regression model, with fixed effects including treatment arm time (categorical), the interaction between treatment and time, anxiety time point (either Time 2 or Time 3), surgical type (stratification variable), and the baseline value of the outcome variable. Differences in mean VAS-A scores between groups at each time point will be presented together with 95% confidence intervals and *p*-values. Continuous secondary outcomes with repeated measurements will be compared using the same model. Hospital length of stay will be compared using the rank-sum test. Patient satisfaction will be compared using an independent sample *t*-test and descriptive statistics will be used to summarise the VRSQ.

#### Missing data {20c}

Missing data will be handled by complete case analysis. Data collection will occur within the same day and thus missing data should be rare.

### Ethical issues {22}

The study, including the participant information and consent form, has been reviewed and approved by the Royal Brisbane & Women’s Hospital Human Research Ethics Committee (RBWH HREC) (HREC/2021/QRBW/74417). Participation is voluntary and decline to participate will involve no penalty or loss of benefits to which the participant is otherwise entitled, and participants may discontinue participation at any time without comment or penalty.

There are possible risks (side effects) associated with the VR intervention ^(38).^ Although rare, side effects may include headache, nausea, vomiting, sweating, fatigue, drowsiness, disorientation, and apathy [33]. Any adverse events that arise, whether related to the study intervention or not, will in the first instance be reported to the clinical nurse manager and to the researchers, who will then submit a report of the event to the HREC.

### Data storage, access, and disposal {19} {27}

Unique project codes will be assigned to participants on entry to the project and stored securely and separately from participant data. During each data collection, research staff will enter participant data into a database. At no time will identifiable study information be reported or made available to persons other than the research personnel.

All electronic data will be stored on a password-protected computer. All paper records will be kept in a locked cabinet in the Principal Investigator’s office. All data will be kept for 15 years following the project’s completion, after which time it will be destroyed or erased from the nominated drive. Only the study researchers will have access to any identifiable data, except where required by law. Data will be reviewed weekly by the researcher for auditing trial conduct.

If the participant decides to withdraw from the project, the researcher will discuss any special requirements linked to withdrawing.

### Plans for communicating significant protocol amendments to relevant parties {25}

When the trial protocol needs to be amended, the main investigators will discuss, communicate, and conclude the revisions. A revised protocol will also be submitted to the Ethics Committee for approval.

### Dissemination plans {31a}

The results of the study will be disseminated to interested parties through publications in an appropriate journal.

## Discussion

Preoperative anxiety is a common condition that adversely impacts approximately 320,000 Australians each year [38]. Unfortunately, preoperative anxiety is frequently overlooked and undertreated. This has become exacerbated with the move to day-of-admission surgery and limited preoperative preparation.

Anxiety should not be overlooked because it has severe effects on patients’ adverse psychological and physiological effects. The psychological effects include a feeling of unease, excessive fear, worry, tension, apprehension, catastrophising, or obsessive thinking [39, 40]. The physiological effects include increased respiratory rate, heart rate, and blood pressure [39, 40]. All of these effects can lead to an increase in the risk of postoperative complications such as pain, need for a greater dose of anaesthesia, delayed wound healing, infection, prolonged recovery, longer hospitalisation, and patient dissatisfaction [12, 41, 42].

There are significant costs associated with these related postoperative complications [43]. However, there is a potential cost saving from preventing or reducing anxiety. Non-pharmacological treatments, like VR, that can be administered by nurses or even self-administered by patients, offer a simple and cheap option for addressing the burden and impact of perioperative anxiety [17].

This current study has several strong features which include the design of a randomised controlled trial (RCT) in accordance with the CONSORT guidelines which is considered the ‘gold standard” in evidence-based research and will provide strong evidence of the effectiveness of using VR on perioperative anxiety compared with non-clinical trial design [44]. In addition, it will be the largest study in adults and the first to screen participants’ level of anxiety using a validated tool—APAIS—before the intervention [28, 29]. Screening for moderate to severe anxiety ensures that the study population requires treatment, which will reduce the dilution of the intervention’s effectiveness, giving a more accurate insight into its clinical relevance. Moreover, this is believed to be the first RCT to combine patient-reported anxiety with the measurement of saliva cortisol level as a biological marker of the stress response.

## Trial status

The trial is ongoing with the first recruited participants on 10 November 2021 and the approximate date of completed this trial will be 11 November 2022.

## Supplementary Information

**Additional file 1.**

## Data Availability

The study principal investigator will have sole access to the final trial dataset. Upon study completion, data will be available from the study principal investigator upon reasonable request after all identifiable information has been removed. All request for data that comply with Australian privacy laws will be accepted.

## Abbreviations

VR: Virtual reality
CONSORT: Consolidated Standards of Reporting Trials
HREC: Human Research Ethics Committee
RBWH: Royal Brisbane & Women’s Hospital
APAIS: Amsterdam Preoperative Anxiety and Information Scale
